# Percutaneous trans-axilla transcatheter aortic valve replacement

**DOI:** 10.1007/s00380-022-02082-3

**Published:** 2022-05-03

**Authors:** Atsushi Sugiura, Mitsumasa Sudo, Baravan Al-Kassou, Jasmin Shamekhi, Miriam Silaschi, Nihal Wilde, Alexander Sedaghat, Ulrich Marc Becher, Marcel Weber, Jan-Malte Sinning, Eberhard Grube, Georg Nickenig, Efstratios I. Charitos, Sebastian Zimmer

**Affiliations:** 1 Department of Medicine II, Heart Center Bonn, University Hospital Bonn, Bonn, Germany; 2 Department of Cardiac Surgery, Heart Center Bonn, University Hospital Bonn, Bonn, Germany

**Keywords:** Transaxillary, Percutaneous, TAVR, Axilla, Distal puncture

## Abstract

The left axillary artery is an attractive alternative access route for transcatheter aortic valve replacement (TAVR) and may provide better outcomes compared to other alternatives. Nevertheless, there remain concerns about vascular complications, lack of compressibility, and thorax-related complications. Between March 2019 and March 2021, 13 patients underwent transaxillary TAVR for severe aortic stenosis at the University Hospital Bonn. The puncture was performed with a puncture at the distal segment of the axillary artery through the axilla, with additional femoral access for applying a safety wire inside the axillary artery. Device success was defined according to the VARC 2 criteria. The study participants were advanced in age (77 ± 9 years old), and 54% were female, with an intermediate risk for surgery (STS risk score 4.7 ± 2.0%). The average diameter of the distal segment of the axillary artery was 5.8 ± 1.0 mm (i.e., the puncture site) and 7.6 ± 0.9 mm for the proximal axillary artery. Device success was achieved in all patients. 30-day major adverse cardiac and cerebrovascular events were 0%. With complete percutaneous management, stent-graft implantation was performed at the puncture site in 38.5% of patients. Minor bleeding was successfully managed with manual compression. Moreover, no thorax-related complications, hematomas, or nerve injuries were observed. Percutaneous trans-axilla TAVR was found to be feasible and safe. This modified approach may mitigate the risk of bleeding and serious complications in the thorax and be less invasive than surgical alternatives.

## Introduction

Transcatheter aortic valve replacement (TAVR) is now a first-line therapy in patients with aortic stenosis, and the femoral artery has been adopted as the primary access point [1, 2]. Nevertheless, the transfemoral approach is not feasible in up to 15–20% of TAVR candidates [3]. Furthermore, vascular complications can be associated with a dismal prognosis. For this reason, numerous efforts have been made to develop alternative approaches [4–6]. The left axillary artery is an attractive alternative access route and may provide better outcomes compared to the other methods, requiring thoracotomy (transapical, direct aortic). Although, the axillary artery is less affected by atherosclerosis, compared to the femoral artery, there remain concerns about vascular complications [7], lack of compressibility, thorax-related complications, and nerve injury through the intervention [8, 9]. Herein, we report our modified transaxillary TAVR approach, namely, “trans-axilla” TAVR where vascular access of the distal axillary artery is gained through the left axilla. This approach may allow us to eliminate the thorax-related complications and offer better compressibility. The puncture technique and vascular management of the puncture site for this procedure are described in the following sections.

## Materials and methods

### Population

Between March 2019 and March 2021, a total of 13 patients underwent transaxillary TAVR via the axilla for severe aortic stenosis at our institution. All patients were considered ineligible for the transfemoral TAVR approach because of severe peripheral artery disease, as assessed by computed tomography imaging and angiography. This study was approved by the institutional ethics committee of the University of Bonn and conducted in concordance with the Declaration of Helsinki. All patients provided written informed consent to the local TAVR registry.

### Trans-axilla TAVR procedure

**Fig. 1:**
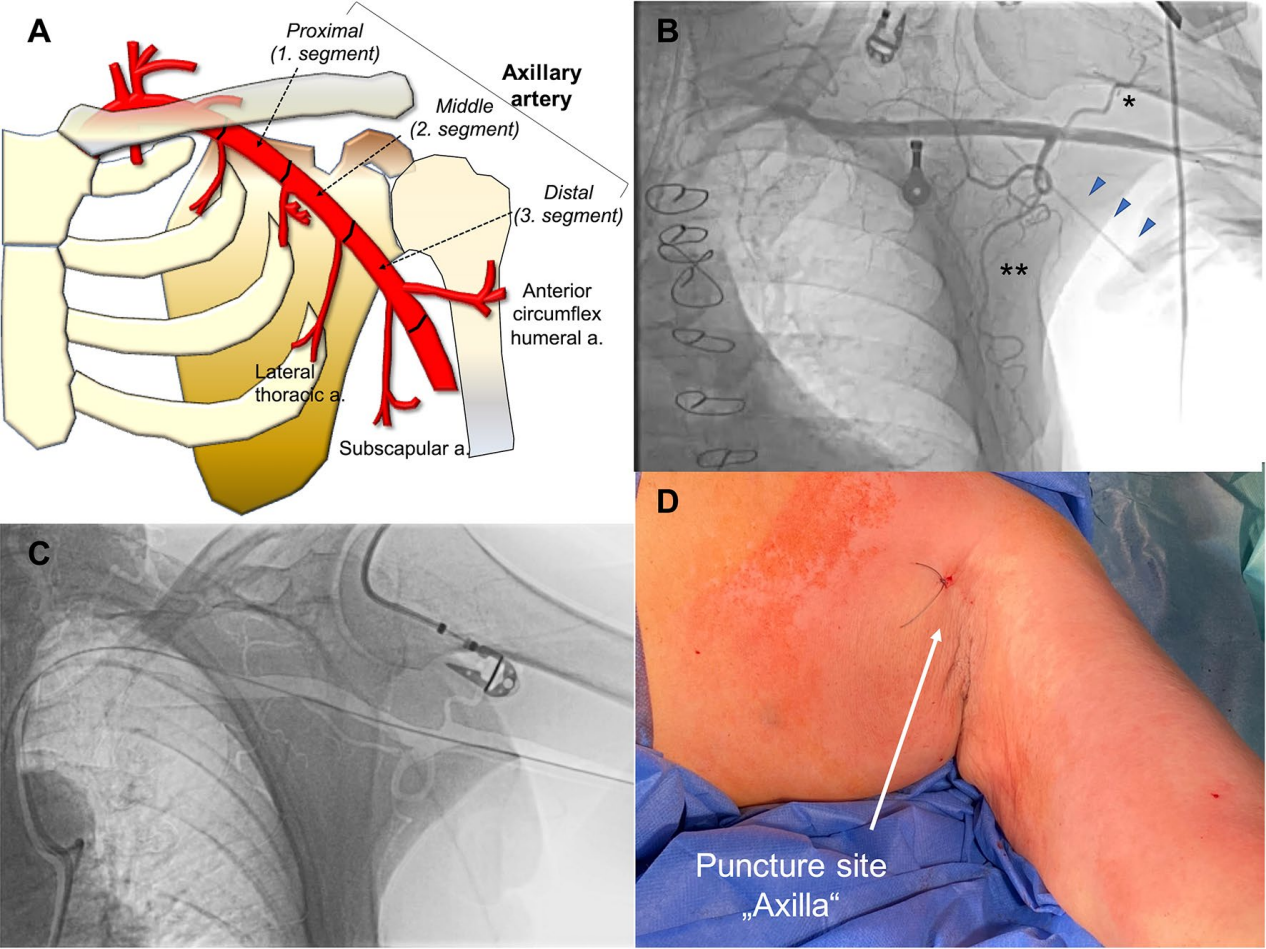
Angiography, schematic illustration, and access site of trans-axilla TAVR **A** The axillary artery begins at the lateral border of the first rib, as a continuation of the subclavian artery. The axillary artery is divided into three segments based on its relation to the pectoralis minor muscle. The first, second, and third segments (i.e., proximal, middle, and distal segments) are medial, posterior, and lateral of the pectoralis minor muscle. The puncture site should be within the third segment of the axillary artery, considering the anatomical relationship to the humeral head. The access point at the third segment of the axillary artery can be compressed against the neighboring osseous structures to control bleeding during sheath removal; **B** angiography of the subclavian and axillary arteries, showing a puncture needle (triangle arrowheads) pointing at the third segment of the axillary artery, which is normally proximal of the take-off of the anterior humeral circumflex artery asterisk and subscapular artery double asterisk; **C** a long J-tip wire is advanced into the axillary artery as a landmark for the puncture and kept in place as a safety measure in case of bleeding complications. A roadmap overlap-view technique as well as ultrasound guidance can be helpful in guiding the puncture. s.; **D** this technique is performed via the “axilla.” The puncture site is located in front of the humeral head, thereby allowing for better compressibility and manual hemostasis if bleeding is observed

The TAVR procedures were performed under general anesthesia. The axillary artery is divided into three segments based on its relation to the pectoralis minor muscle. The first, second, and third segments are medial, posterior, and lateral to the pectoralis minor muscle (i.e., proximal, middle, and distal segments). The puncture was performed within the third segment of the axillary artery, considering that the anatomical relationship with the humeral head allows physicians to compress the puncture site after the procedure efficiently. Furthermore, the puncture should be performed proximal of the take-off of the anterior humeral circumflex artery and subscapular artery (Fig. 1A), given that the median nerve generally transverses in front of the transition between the axillary and brachial arteries. If available, a roadmap overlap-view technique as well as an ultrasound guidance can be helpful for targeted axillary artery puncture.

First, a 6F sheath was placed into a femoral artery, and a diagnostic JR 4.0 was placed in the subclavian artery. Contrast dye was injected through the catheter to visualize the access route, and a long (260 cm) J-tip wire was advanced into the axillary artery. Second, using fluoroscopy with a landmark of the inserted wire, the distal axillary artery was punctured through the left axilla (Fig. 1B). A roadmap overlap-view technique (Fig. 1C) as well as an ultrasound guidance proved to be helpful for targeted axillary artery puncture. This technique is performed via the “axilla” (Fig. 1D). The puncture site is located in front of the humeral head, thereby allowing for better compressibility and manual hemostasis if bleeding is observed.

A “pre-suture” using two ProGlide^®^ devices as the closure system was utilized in most patients (10 out of 13 patients). A MANTA vascular closure device was applied in the remaining three patients. While placing the pre-suture ProGlide system, temporarily pulling the J-tip landmark wire back to the subclavian artery was recommended in order to avoid trapping the wire between the ProGlide^®^ system and the vessel wall. A 9F sheath was subsequently inserted into the axillary artery.

**Fig. 2:**
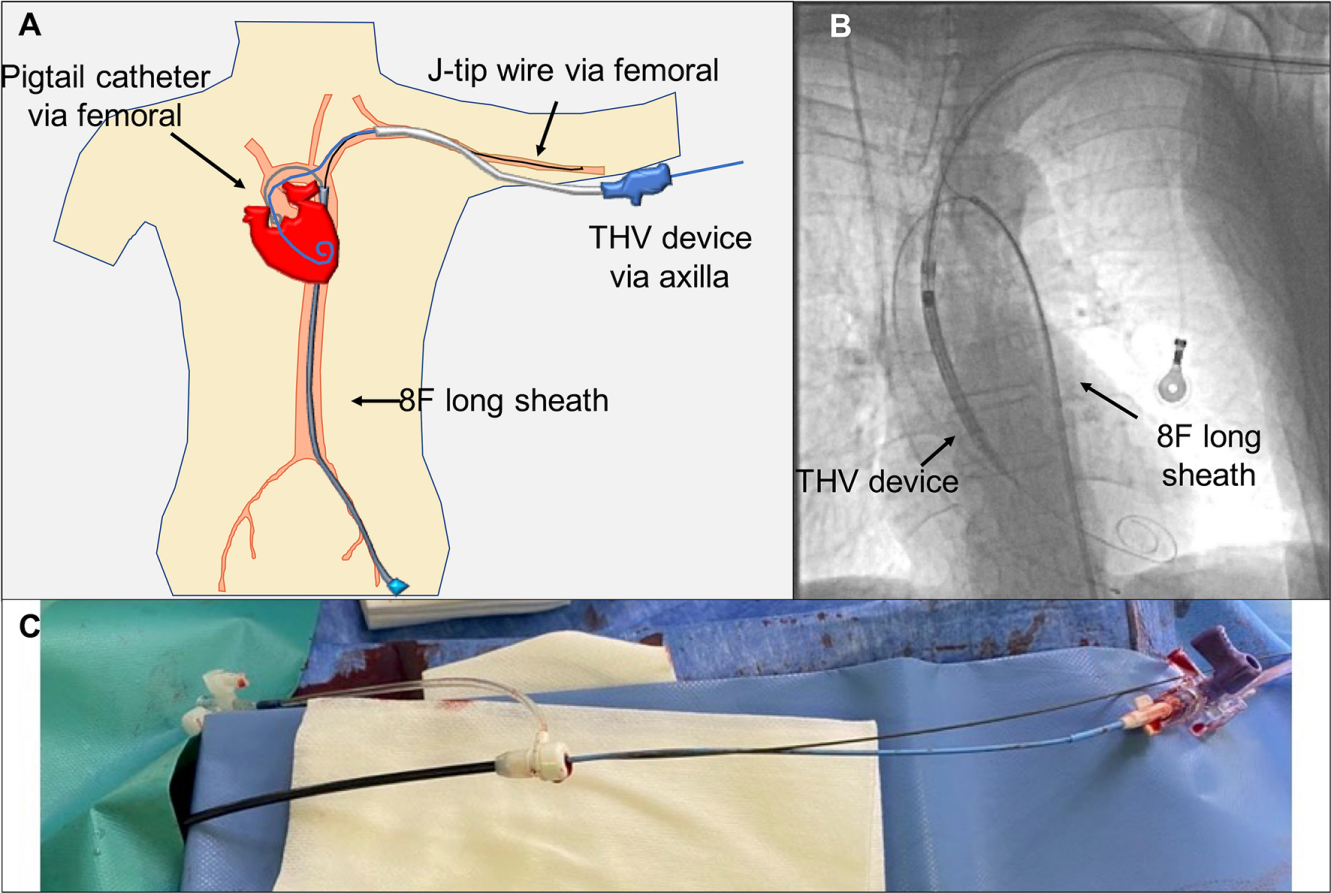
Schematic illustration of trans-axilla TAVR set-up shown is the set-up for a trans-axilla TAVR. A pig-tail catheter is placed through an 8F long sheath via the femoral artery for the injection of dye. Also, a safety wire is placed in the axillary artery through the femoral sheath.

**Fig. 3:**
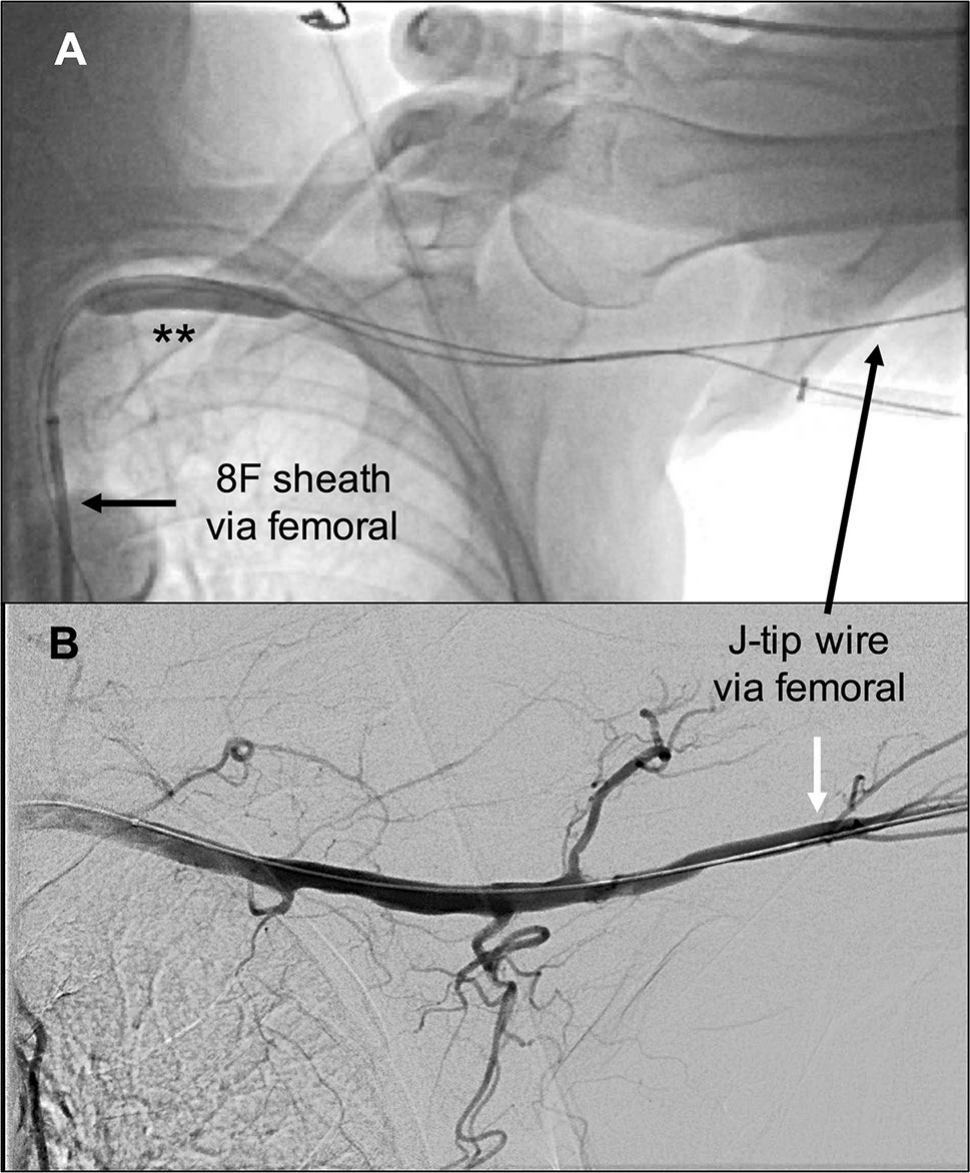
Retrieving the THV sheath and vascular management. **A** An angioplasty balloon double asterisk and the 8F long sheath are advanced into the subclavian artery. A balloon blockade is performed during retrieval of the THV sheath, in order to minimize the bleeding from the puncture site. A J-tip wire via the femoral artery remained in the axillary artery to secure an access route for stent implantation, in case of significant bleeding, perforation, or flow-limiting dissection; **B** after the two sutures of the ProGlide^®^ system are tied down, control angiography is performed using the 8F-80 cm sheath via the femoral artery. The access point at the third segment of the axillary artery can be compressed against the neighboring osseous structures to control bleeding during sheath removal

The short femoral 6F sheath was exchanged for an 8F long sheath. A length of 70–80 cm allowed to maintain a safety wire in the left axillary artery while simultaneously advancing a 5F pig-tail catheter for contrast dye injection to aid valve placement. The tip of the sheath was placed at the transition of aortic arch to the descending aorta (Fig. 2). A stiff guidewire was then inserted via the left axillary artery into the left ventricle and the 9F sheath was exchanged for a 14–16F guiding sheath, depending on the type of transcatheter heart valve (THV) used. After the THV implantation, an angioplasty balloon was advanced into the proximal subclavian artery via the femoral access, while the THV guiding sheath was carefully pulled back. The 8F long sheath was carefully advanced into the left subclavian artery (Fig. 3A). After removal of the THV guiding sheath, the angioplasty balloon was inflated to minimize antegrade blood flow while the J-tip wire (via the femoral artery) remained in the axillary artery to secure an access route for potential stent implantation, in case of significant bleeding, perforation, or flow-limiting dissection (Fig. 3B). After the two sutures of the ProGlide^®^ system were tied down, control angiography was performed through the 8Fcm sheath via the femoral artery. Out of 13 patients, the MANTA vascular closure devise was utilized in three patients instead of the Proglide system.

### Periprocedural antithrombotic regimen

All patients were loaded with 500 mg aspirin intravenously and received unfractionated heparin 70 IU/kg body weight at the beginning of the procedure. Activated clotting time was measured after 15 min and additional heparin administered if required. During the procedure, activated clotting time was maintained at more than 250 s. Heparin reversal with protamine was performed at the end of the procedure. After the procedure, all patients received continuous intravenous unfractionated heparin at 100 IU/h for prevention of deep vein thrombosis. In patients with indications for permanent oral anticoagulation, oral anticoagulation was stopped before the procedure, and bridging with intravenous heparin was performed during the first 48 h after the procedure.

### Endpoint

The primary endpoint for efficacy was device success. Secondary endpoints were 30-day major adverse events (i.e., death and disabling stroke) and periprocedural complications. These endpoints were assessed according to the VARC2 criteria [3].

### Statistical analysis

Categorical variables are reported as percentages and continuous variables are reported as the mean ± standard deviation (SD) or as medians and interquartile ranges (IQRs), as appropriate.

## Results

### Study participants

Overall, the study participants were at advanced age (77 ± 9 years old) and 53.8% were female, with an intermediate risk for surgery (STS risk score: 4.7 ± 2.0%) (Table 1). The mean transvalvular aortic pressure gradient was 39.0 ± 21.8 mmHg, and the aortic valve area was 0.73 ± 0.20 cm^2^. In all patients, trans-axilla TAVR was performed using the left axillary artery. At the time of the preprocedural computed tomography imaging, the average diameter was 7.6 ± 0.9 mm for the first segment of the axillary artery, 6.7 ± 1.0 mm for the second segment, and 5.8 ± 1.0 mm for the third segment. Mild calcification was observed in 10 (76.9%) patients, while none of the patients had severe calcification in these arteries.

**Table 1:** Baseline characteristics

Age, years	77 ± 9
Sex female	7 (53.8)
Body surface area, kg/m^2^	1.76 ± 0.20
Coronary artery disease	12 (92.3)
Atrial fibrillation	2 (15.4)
Chronic obstructive pulmonary disease	3 (23.1)
STS score, %	4.7 ± 2.0
Creatinine, mg/dl	0.8 (0.5, 1.3)
Left-ventricular ejection fraction, %	56.9 ± 11.4
Aortic valve area, mm^2^	0.73 ± 0.20
Mean aortic valve gradient, mmHg	39.0 ± 21.8
MR moderate or more	3 (25.0)
Right ventricular systolic pressure, mmHg	35.9 ± 17.0
Annulus diameter, mm	24.2 ± 2.6
LCA height, mm	13.8 ± 2.0
RCA height, mm	18.2 ± 2.8
Proximal subclavian artery, mm	10.3 ± 1.8
Distal subclavian artery, mm	6.8 ± 0.9
Proximal axillary artery, mm	7.6 ± 0.9
Mid axillary artery, mm	6.7 ± 1.0
Distal axillary artery, mm	5.8 ± 1.0
Severe calcification	0
Severe tortuously	0

Values are the mean ± SD or *n* (%)

*LCA* left coronary artery, *RCA* right coronary artery

### Procedural outcome

Device success was achieved in all patients (Table 2). There were no periprocedural deaths, emergency surgery, or 30-day major adverse events (i.e., death and disabling stroke). They exhibited no subsequent neurological complications (e.g., restricted arm movement). Moreover, no major vascular complications occurred. One patient underwent the procedure with a puncture in the second segment due to the small vessel size of the third segment (4.1 mm). Stent-graft implantation was performed at the puncture site in five (38.5%) patients with persistent extravasation after the vascular closure device, whereas there was no significant difference in the vessel diameters between patients with and those without stent-graft implantation (first segment: 7.5 ± 1.2 mm vs. 7.6 ± 0.8 mm, p = 0.78); second segment: 6.7 ± 1.1 mm vs. 6.7 ± 1.0 mm, *p* = 0.37; third segment: 5.7 ± 1.1 mm vs. 6.1 ± 1.0 mm, *p* = 0.37). Otherwise, minor periprocedural bleeding was successfully managed with the administration of protamine and manual compression. Also, no pneumothorax or hemothorax were observed. Of the two patients who received multiple blood transfusions, one had a baseline hemoglobin level of 9.0 g/dl due to macrocytic anemia, which declined to 7.3 g/dl after the procedure. The other patient had gastrointestinal bleeding after the procedure, which required transfusion of 4 packs of red blood cell transfusion. Therefore, they were classified as having major bleeding and life-threatening bleeding, respectively. In one patient, the puncture site was surgically corrected because of the J-tip wire was trapped by the ProGlide^®^ knot loop. All patients were extubated in the hybrid operating room.

**Table 2:** Procedural findings

General anesthesia	13 (100)
THV devices used	
Evolut R/PRO	12 (92.3)
Sapien 3	1 (7.7)
Predilatation	1 (7.7)
Postdilatation	2 (15.4)
Contrast volume, ml	134.8 ± 39.2
Fluoroscopy time, min	29.7 ± 13.8
Procedural time, min	107.3 ± 46.8
Successful THV implantation	13 (100)
Conversion to surgery	0
Closure devices used	
ProGlide	10 (76.9)
Manta	3 (23.1)
Stent implantation in the axillary artery	5 (38.5)
Bail-out surgical cutdown	1 (7.8)
30-day outcomes	
Mortality	0
Disabling stroke	0
Myocardial infarction	0
Major bleeding	1 (7.8)
Major vascular complication	0
Multiple blood transfusions	2 (15.6)
Acute kidney injury	0
Paravalvular leakage moderate or more	0

Values are the mean ± SD or *n* (%)

*THV* transcatheter heart valve

## Discussion

In this study, we describe a modified trans-axilla TAVR and its procedural results.

Our main findings are as follows:

1. Trans-axilla TAVR with complete percutaneous management is feasible and safe in patients who are not eligible for a transfemoral approach, with a 100% success rate for device implantation.
2. There were no 30-day major adverse events. Moreover, no thorax-related complications, hematomas, or nerve injuries were observed.
3. In addition to the TAVR access site, only one femoral 8F sheath is required for contrast dye injection and to maintain a safety wire inside the axillary artery.

Transaxillary TAVR is increasingly popular among interventional cardiologists for patients ineligible for transfemoral arterial access [4–6]. In general, the arteries of the upper extremities are more likely to be free of atherosclerosis and calcification, even if the iliofemoral arteries are diseased [10]. Axillary access offers less invasiveness and several advantages over the transapical or transaortic approaches, which include a lower risk of periprocedural bleeding, swift recovery after the procedure, and reduced mortality [11]. Given the risk of major bleeding or wound complications through surgical cut-down approaches [12], fully percutaneous management is a safe, feasible, and attractive way to perform transaxillary TAVR [13]. Nevertheless, there still remains concerns for this procedure with regard to periprocedural bleeding, hematoma, pneumothorax, and hemothorax, as the proximal part of the axillary artery is surrounded by relatively soft tissues and is located on the thorax [14]. Moreover, difficulties in effecting hemostasis, given the lack of compressibility of this artery due to the surrounding bone structures, may explain why transaxillary access is often not selected.

Herein, we have illustrated a modified approach to this technique, namely, trans-axilla TAVR. The major advantage of our technique is that it allows the artery to easily be compressed at the puncture point, as is in the transfemoral approach. Very much like transfemoral TAVR, it allows for a complete percutaneous access of the THV and provides compressibility owing to the presence of the humeral head just behind the puncture site. Herein, successful implantation of a THV was achieved in all patients, with no incidence of major bleeding complications related to the access site. Not surprisingly, the distal section of the axillary artery was smaller than the proximal part, however, no major vascular complications occurred in the current study. In line with our findings, Tayal et al. reported that the axillary artery was normally large enough to accommodate a sheath with up to 18 French [15] and yielded vascular complications of 0%. Another advantage of our technique is to be able to eliminate the risk of pneumothorax or hemothorax. Coupled with earlier knowledge [16–18], our findings suggest that trans-axilla TAVR serves as a safe alternative for patients who are ineligible for transfemoral arterial access.

Patient selection is a critical step for successful trans-axilla TAVR. One important concern of this technique might be related to the diameter of the axillary artery. Although the third segment of the axillary is preferable for the puncture site, in the current cohort, one patient underwent a trans-axilla TAVR with a puncture in the second segment of the axillary artery owing to the small diameter in the third segment (4.1 mm). In patients with a history of coronary artery bypass graft using the left internal mammary artery, the diameter of the artery at the take-off of the mammary artery should be greater than 5.5 mm [19]. The presence of an atrial-venous shunt for dialysis on the TAVR access is regarded as a relative contraindication, although we believe that a trans-axilla TAVR could be performed with a large enough diameter of the axillary artery. Also, severe calcification, excessive tortuosity, steep angulation of the artery should be precluded. As in trans-subclavian TAVR, both the left and right axillary approaches can be used, whereas the left is preferred due to better alignment with the native annulus. Also, the trans-axilla TAVR can be performed without any femoral access but with bilateral radial accesses [20]. Moreover, our modified approach “trans-axilla TAVR” with percutaneous access site management could also be performed under local anesthesia instead of general anesthesia. Further investigations are needed to validate our preliminary findings and explore these issues.

Another concern is nerve injury associated with interventions. Even though, in general, nerve injuries after interventions are rare and usually transient [8], caution should be cared for to prevent significant functional impairment. In the present study, no nerve injury or hematoma occurred after the intervention. An appropriate puncture site is an essential factor to prevent nerve injury. The previously reported higher rate of nerve complications is likely due to a more distal puncture site as we performed in the current management [9]. The puncture should be performed proximal of in the proximal to the take-off of the anterior humeral circumflex artery and subscapular artery (Fig. 1), given that the median nerve generally transverses in front of the transition between the axillary and brachial arteries [21, 22]. In addition to fluoroscopic guidance using a roadmap overlap-view technique, ultrasound guidance will be helpful for targeted axillary artery puncture. Also, optimal vascular management at the puncture site (i.e., safety wire, stent-graft implantation) is paramount since nerve injury more often results from hematoma or pseudoaneurysm formation and less commonly through direct damage from the needle puncture or manual compression [8].

The major limitations of this study are derived from the retrospective nature with a small sample size. Patients were not randomized to several alternative approaches. Furthermore, although no patients reported brachial plexus injury, longer follow-up data beyond 30 days were not evaluated. Our preliminary findings should be validated in further studies with larger cohorts and long-term clinical outcome data.

## Conclusions

The present analysis showed that percutaneous trans-axilla TAVR was safe and feasible, with no thorax-related complications, hematomas, or nerve injuries. This modified approach may mitigate the risk of bleeding and serious complications related to the thorax.
